# Transitional Justice after the COVID-19 Pandemic

**DOI:** 10.3390/ijerph191912388

**Published:** 2022-09-29

**Authors:** Bruno Rodríguez Reveggino, Ángel Becerra-Bolaños

**Affiliations:** 1 Inter-American Court of Human Rights, San José 6906-1000, Costa Rica; 2 Faculty of Law, Universidad Tecnológica del Perú, Arequipa 04001, Peru; 3 Department of Anesthesiology and Intensive Care Unit, Hospital Universitario de Gran Canaria Doctor Negrín, 35010 Las Palmas de Gran Canaria, Spain; 4 Department of Medical and Surgical Sciences, Faculty of Health Sciences Universidad de Las Palmas de Gran Canaria, 35001 Las Palmas de Gran Canaria, Spain

**Keywords:** transitional justice, COVID-19, human rights, pandemic, public health, reparations

## Abstract

The COVID-19 pandemic has been a real challenge for health systems and public policies. Both the pandemic and the measures taken to mitigate it have affected the freedoms and rights of the different sectors of society, especially the most vulnerable ones, and have increased the already existing structural inequalities. Consequently, the pandemic must be analyzed from the perspective of human rights. Transitional Justice (TJ) has proven to be useful after conflict situations, helping societies to confront abuses perpetrated and to find solutions for the future, as well as repairing damages that have arisen as a consequence of these conflicts in different areas. Thus, TJ processes have been successfully used after armed conflicts and during peace negotiations, to respond to abuses perpetrated in consolidated democracies, and even after environmental crises. Therefore, the creation of a “Truth and Reconciliation Commission for the COVID-19 pandemic”, which launches the TJ processes of truth, justice, reparation and guarantees of non-repetition can help to find solutions to conflicts arising from the pandemic in a simple way. In addition, it would establish the foundations to prevent the violation of human rights in similar situations to come.

## 1. Introduction

The COVID-19 pandemic has been a challenge for medical emergencies, as it has constituted the greatest pandemic in the contemporary era. Public health policies have been examined and the shortcomings of the systems in a challenging situation hitherto unknown have been revealed. Thus, the overload of health resources even led to making controversial decisions, such as the decision not to resuscitate those severe patients who suffered cardiorespiratory arrest in intensive care units without taking into account the opinion of patients or relatives [1]. In addition, the lack of personal protective equipment and PCR tests at the beginning of the pandemic was evident [2]. In addition to the lost human lives having reached the millions [3], the life expectancy of the population in some territories of the planet has been affected [4].

Due to the pandemic mitigation measures taken by various countries, people have seen their rights and freedoms directly or indirectly affected. Thus, the already existing structural inequalities have been accentuated [4]. Limitation of freedoms has followed more an economic motivation than the true motivation of mitigating the spread of the disease. The pandemic has put our society and jurisprudence in a stressful situation. The various and often inconsistent regulations that sought to balance the needs of public health control with the economic imperatives were difficult for the executive powers to explain and difficult to understand for the citizens that suffered from constant fear for its health and well-being [5]. Thus, COVID-19 is not only a public health issue, but also a human rights issue [6]. The Inter-American Court of Human Rights has stated that the problems and challenges that arise from the pandemic must be addressed from the perspective of human rights, respecting the international obligations of the countries concerned [7].

For several decades, international human rights law and international humanitarian law have known violations that, due to their massiveness or impact, require particular mechanisms to respond to the victims. Traditionally it has been understood that Transitional Justice (TJ) is a useful tool to deal with an armed conflict, a dictatorship, or an authoritarian regime. According to Bickford, TJ is a set of judicial and non-judicial mechanisms to deal with “past human rights abuses, mass atrocity, or other forms of severe social trauma, including genocide or civil war, in order to build a more democratic, just, or peaceful future” [8]. In many cases, this tool has demonstrated its usefulness in situations of armed conflict as the case with the Chapultepec peace accords in El Salvador, whose aim was “ending the armed conflict through political means in the shortest possible time, promoting democratization of the country, guaranteeing unrestricted respect for human rights and reunifying society” [9]. The same happened with the Truth and Reconciliation Commission in Peru, which establishes that “for reconciliation to be viable is necessary […]: the overcoming and definitive resolution of the conflict, the critical discussion of the ideas of reconciliation among different sectors […] and the adoption of State policies that meet the demands of civil society [, which] implies a profound reform” [10]. In these two examples the human rights violations were consequences of armed conflicts such as: torture, forced disappearances, sexual violence, extrajudicial killings, illegal and arbitrary detentions, among others [11,12].

TJ has also been used in the framework of peace negotiations. The most paradigmatic case is the peace accords signed in 2016 by the FARC and Colombia [13], where ‘‘transitional measures of memory reconstruction, reparation, land restitution, demobilization, alternative sentences, and a comprehensive peace negotiation process’’ were combined [14,15].

However, TJ processes have also been applied in established democracies, such as in Canada, where these mechanisms have been used to respond to abuses against children who have been sexually or physically abused [16]. The application of TJ has also been proposed to respond to environmental crises, through the application of amnesties, accountability measures, truth commissions, reparations, and institutional reform [17].

Taking into consideration the diverse scope of its application, TJ may be understood as a framework “for confronting past abuse as a component of a major political transformation” [8]. Therefore, this conceptual article proposes that it is legally viable, under the international law, to deal with the COVID-19 pandemic aftermath through a “Truth and Reconciliation Commission for the COVID-19 pandemic” (TRC-COVID19). The TJ framework will serve to determine the scope and content of said Commission. Therefore, a roadmap can be proposed from international law to establish a TJ process for the post-pandemic reality. The objective is not analyzing the management of the pandemic from a national perspective, nor evaluating each of the measures that countries took to mitigate the spread of the virus from the epidemiological or medical field. This would require more research and reflection, including these aspects from the perspective of domestic law. However, it is necessary to start planning from the TJ point of view the aspects that will have to be considered so that the possible injustices that occurred during the pandemic do not occur again in similar future scenarios.

## 2. Transitional Justice in Light of International Law and Its Application to the Post-Pandemic Situation

TJ mechanisms are very diverse, both in their nature and dimensions. They could be part of large or small processes. From the aftermath of mass human rights violations such as in Rwanda, passing the end of a system such as with the apartheid in South Africa, to deal with child abuses in Canada, are all considered TJ processes. In addition, the nature of TJ could be both judicial or extrajudicial in nature. Their configuration largely depends on the societies in which they are applied [18]. Even though there is no homogeneous definition of TJ [8], there is a consensus that these processes serve societies to confront the abuses perpetrated on a large scale and solve problems for the future. In this way, its main objective is that “those responsible be held accountable for their actions, serve justice and achieve reconciliation” [18].

The United Nations (UN) General Secretary identified four components common to all TJ processes: truth, justice, reparation and guarantees of non-repetition [18]. There are no paradigmatic or static standards on the interaction of these components and the balance of each one depends on the social, political, and legal realities they face [15]. Depending on the peculiarities of each case, the components of TJ can vary considerably as long as international standards are met.

In its own semantic structure, TJ finds its two “defining characteristics” [8]. First, the concept of “justice”. Therefore, it is not necessary to create a special jurisdiction to deal with human rights violations that can be evidenced throughout the TJ process, being that the use of the ordinary jurisdiction is sufficient and pertinent. The judiciary of each country should respond without the need for the creation of a special jurisdiction. An example of a special jurisdiction created specifically to try and punish those responsible for human rights violations is the Special Jurisdiction for Peace in Colombia, which arose as a result of the peace agreements with the FARC in 2016 [19]. Although in the traditional concept of TJ there has been an emphasis on the issue of justice, even traditional authors such as Louis Bickford consider that it is possible that the need for justice was covered from well-known reparation programs or truth mechanisms. such as truth-seeking mechanisms [8]. Of course, the findings of the TRC-COVID-19 must be made available to the pertinent jurisdictional instances to be treated in accordance with the current legal framework.

Second, concerning the “transitional” concept, the main argument that could be used against the use of the TJ regulatory framework for the post-pandemic stage is that it is not a transition situation in the strictest meaning of the word. We are not facing a post-conflict or post-dictatorship scenario. However, this pandemic has been a turning point, evidencing large-scale human rights violations, such as the lack of adequate access to health systems, or restrictions on fundamental rights such as the right of mobility or meeting. The limitations of health systems in “the end-of-life approach” or the limitation of resources for those patients most seriously affected by the disease have also been evidenced. Inequality in terms of race or social status in access to health care or to the vaccine has been also observed [20]. However, it is not necessary to delve into the attribution of responsibility of the state in these human rights violations, since this would require a greater and more detailed analysis. It is possible to begin repairing the damage perpetrated by applying the concepts of TJ in a generalized way in the current context, without the need to go into each specific case.

Pablo De Greiff, the first Rapporteur of the UN for the promotion of justice, truth, reparation and guarantees of non-repetition, affirms that there is a consensus that the term TJ refers to the “set of measures implemented by several countries to deal with massive human rights violations” [21]. Although these measures may be varied, they must be comprehensive in addressing past injustices and not be “items on a random list” [21]. In this line, it is necessary to conceptualize TJ as a holistic system. Therefore, initiatives need to be concentrated or channeled from a coherent and holistic mechanism following De Greif′s methodological proposal, considering the experiences of societies that have experienced long-lasting conflicts, where there have been many “unofficial” initiatives, without obtaining optimal results [22]. Although these initiatives have been positively recognized, there have been also critics of the fact that they have not been made into official mechanisms [23]. To this extent, a mechanism such as the TRC-COVID-19, integrated with the international regulatory framework, is pertinent so that it exercises the role of rector of the TJ process, in an official manner and from the state, but also in accordance with the citizenship′s will.

## 3. Scope and Application: A Roadmap for the “Truth and Reconciliation Commission for the COVID-19 Pandemic” (TRC-COVID-19)

In this section a TRC-COVID-19 roadmap is proposed. Its composition (number and characteristics of each component), as well as the time frame in which it will operate will remains to be defined by each country. The following items are proposed in this article: (a) substantive scope, (b) structural scope, and (c) teleological scope or purposes.

### 3.1. Substantive Scope

The pandemic affected the rights and freedoms of all social strata. However, it disproportionally affected people in situation of vulnerability. In this article, we use the Declaration “COVID-19 and Human Rights”, issued by the Inter-American Court on 9 April 2020 [7], as a framework to determine the scope of the TRC-COVID-19. The following substantive rights fall under the scope of it:

Restriction and suspension of rights, and the state use of force. Many of the measures taken by states to mitigate the spread of the virus were related to lockdowns and restrictions on the free movement of persons. In many cases the expansion of police and military presence caused a disproportionate use of force and other human rights violations. Human rights violations include arbitrary arrests and detention, unlawful use of force, torture and other ill-treatment, forced evictions, illegal expulsion of refugees and migrant workers, and discriminatory policing. For instance, 13 persons allegedly died in Lima, Peru, “trying to escape police who raided a nightclub violating coronavirus restrictions” [24]. In Spain a young man and his mother were pinned down and beaten by the policy in the context of the lockdown [25]. Furthermore, in many cases, freedom of expression has been illegally and legitimately restricted. The UN Special Rapporteur on the Promotion and Protection of the Right to Freedom of Opinion and Expression, confirms that the response to the COVID-19 virus has been used as a “pathogen of repression” [26]. In some countries that have legislation that represses freedom of expression such as China, Cuba, Indonesia, and Nicaragua the pandemic has provided a new context to shut down independent reporting or dissident voices under the guise of protecting public health [27]. Regarding women′s rights, a rise on gender-related violence has been reported due to the lockdowns [28]. Emerging data shows an increase in calls to domestic violence helplines in many countries since the outbreak of COVID-19. Data in Latin America shows an increase of 80%, 76% in Colombia, 70% in Chile and 39% in Argentina [29]. Additionally, according to the Eurobarometer, three out of four women (77%) in the EU think that the COVID-19 pandemic has led to an increase in physical and emotional violence against women [30]. In addition, there has been an important impact on the economic rights of women. Women are more likely to be unpaid; or are on insecure, temporary, and short-term contracts; and/or are concentrated in low-wage, part-time, informal work, and in the service and retail industries [31]. According to the UN, the pandemic generated a setback of more than a decade in the levels of labor participation of women in Latin America [32]. With regards to corruption and human rights, the pandemic has increased corruption risks and, in many cases, served as a catalyst for corruption. On the one hand, there has been concerns about how to maximize the transparency of treatments and vaccines for COVID-19 that are approved or are under evaluation This includes vaccines allocation and global and local equal distribution, since there are many allegations on inequality from administrations leaving disadvantaged individuals and countries behind [33]. On the other hand, the UN has highlighted the importance of preventing corruption in in the allocation and distribution of emergency economic rescue packages in the context and aftermath of the COVID-19 pandemic [34]. Concerning the impact on people in situations of vulnerability, i.e., disabled people, elderly, prisoners, children, migrants, and LGBTIQ persons, the United Nations High Commissioner on Human Rights has stated “the free movement of persons have increased the risk of isolating particularly vulnerable groups, including older persons and persons with disabilities, with consequences for their mental health and physical well-being” [35]. In some countries such as the United States, it has been also reported how Afro descendants and Hispanic communities have been disproportionately affected by the access to adequate health services [36]. In addition, in Belgium, Bulgaria, Cyprus, France, Greece, Hungary, Italy, Serbia, Slovakia, Romania, Spain and the UK, Amnesty International reported racial violence in the framework of lockdowns [37]. It is also relevant to highlight the situation of people with disabilities. Whereas on the one hand persons with disabilities experience higher mortality rates, on the other, they face significant barriers in access to health care. The UN High Commissioner on Human Rights has reported discriminatory medical protocols. For instance, there has been blanket “Do Not Attempt Resuscitation” (DNAR) notices imposed on them without their consent [38].

### 3.2. Structural Scope

Considering that the elements of the TJ process are truth, justice, reparation and guarantees of non-repetition, already depicted in the previous section, it is relevant to establish the following mechanisms:

1. Truth and Reconciliation Commission for the COVID-19 pandemic, TRC-COVID-19: Collegiate body that will lead the TJ process. Its features will be addressed by the future comprehensive TJ plan. However, it can be pointed out that it would be necessary to include national and international personalities, with gender parity and ethnic balance, ensuring the participation of members of indigenous and peasant communities, as well as the representation of the professional and social strata mainly affected by the pandemic. A report would be presented within a reasonable period of time that allows addressing the elements of truth, justice, reparation and guarantees of non-repetition.
2. Reparations program: In international law, reparation measures are not only economic, but also include a variety of remedies, such as satisfaction, restitution of rights, rehabilitation and guarantees of non-repetition [39]. Satisfaction measures may include, for example, erecting a monument in favor of the victims. Therefore, it is first necessary to establish the potential victims in the context of the pandemic and to whom these reparations will have to be directed. This group should include those patients who died or who suffer sequelae secondary to the disease itself, as well as their relatives due to the economic or emotional consequences they may have suffered. It is also necessary to include in this group of victims those chronic diseases’ patients whose treatment has been postponed during the contingencies, as well as oncologic patients who have suffered a delay in receiving surgery or chemotherapy [40,41]. This group should also include health professionals who have seen their workload increase and their quality of life reduced during the pandemic, and who are suffering from ailments related to physical and mental health as a result of the situation. Even though governments tried to guarantee the availability of supportive systems for professionals and their families [42], health professionals showed significant levels of mental disorders during the COVID-19 pandemic [43], similar to those detected in previous epidemic situations [44]. This is indicative of the necessary measures not having been taken to mitigate stress in health workers despite former experiences.
3. Given the nature and magnitude of the problem, and also the massive number of potential victims (fatal and non-fatal), reparation measures must address this reality [45], and should not be solely economic measures. It is not the intention that the State be embezzled to care for the victims. For this reason, a measure of satisfaction widely included in reparations programs is the request for public forgiveness by the authorities. Likewise, it is of cardinal importance that the reparation measures are followed by measures that guarantee non-repetition. For this, it would be interesting for the TRC-COVID-19 to perform a subsequent follow-up, ensuring that consensual measures have been carried out, using mechanisms that already exist in international law [46].
4. Technical Committee of reform package to guarantee non-repetition: Technical and scientific collegiate body that will elaborate a comprehensive plan to adopt the necessary legislative measures. These measures with have a social, cultural, administrative, and legal impact, among others. The purpose of these measures is to ensure that the events that already happened do not occur again as consequence of deficiencies related to the lack of infrastructure, deficiency of human resources or limitations in the legal framework. To ensure the “non-repetition”, it will be also necessary to carry out a subsequent follow-up of compliance with the reforms and their temporary maintenance.

### 3.3. Teleological Scope or Purposes

Since the consolidation of the international regime for the protection of human rights, there has been controversies about how to deal with situations of massive violations of human rights. The international legal framework allows the use of mechanisms of TJ, whose purpose goes beyond justice in particular cases, seeking to rebuild the broken social structure and prevent similar situations from happening again. Zalaquett argues that the ethical dilemma for societies after a conflict (or a pandemic, in the case that concerns us), fluctuates between being able to balance certain ethical imperatives with various political constraints [47]. Likewise, De Greiff states that the principles that should guide the TJ processes are the following [21]:

- Recognition of the victims: not only as victims but also as subjects of law. The objective of the reparation measures is not only to repair the suffering caused by the aforementioned victims of the pandemic, but also to restore their ability to exercise their rights as citizens.
- Civic trust: with the use of the legal system of justice. The purpose is effectively to obtain justice, for which an expectation of shared normative commitment must be established. The application of the TJ will increase the trust of the victims in the public health systems and in the executive powers in an eventual future situation of pandemic.
- Reconciliation: describing a state in which social relations are characterized by trust based on the normative framework. Thus, society will increase its trust in institutions and future controversial decisions will be better accepted and followed.
- Democracy and rule of law as an expression of individual autonomy and as a means for citizens to understand justice through the law. In addition, for the TJ measures to be valid, they should be adopted through democratically elected representatives, reflecting the popular will. Without analyzing any particular government model, TJ could contribute to achieve the ideals of democracy and also its modern attributes which are free and fair elections, universal participation, civil liberties, and responsible government [48]. Moreover, it aims to foster the rule of law as restriction of the arbitrary exercise of power.

To these principles raised by De Greiff, we would like to include the following:

- Transparency: active engagement of civil society to the TJ processes, truth commission, information and communication strategies promoting an active participation of victim groups. Thus, in an eventual future pandemic situation, the population will be involved in decision-making taken processes.
- Solidarity: as the most disadvantaged of the social strata suffered greater damage during the pandemic, with a greater deterioration of their economy and their quality of life. For this reason, it is important that the actions taken during the exercise of TJ in this context take these consequences into account and that reparation measures are preferably aimed at those who suffered the worst consequences.
- Diverse representation: these processes must be led by groups made up of people who are not normally represented (racial, ethical, social minorities, LGTBIQ persons), who have suffered harm to their rights and freedoms during the pandemic.

## 4. A Way towards the “New Normal”

According to Oona Hathaway, large system crises create limited windows of opportunity to make large changes in the system [49]. In the past, TJ has been used to reinforce human rights in a particular window of opportunity. The authors strongly believe that the aftermath of COVID-19 provides said window of opportunity [50], and the TJ framework is a useful tool to achieve it. Human rights violations do not occur in isolation. Context is key to understand why and how these violations were possible. The “normality” in which the pandemic occurred has proven to have some deficiencies that allowed the human rights abuses to happen. It is highly believed that the crisis will exacerbate structural inequalities.

Therefore, the use of the international TJ framework is convenient in the post pandemic context. Not only were various human rights directly affected, but also poverty and inequality have worsened in this new normality. As the UN High Commissioner for Human Rights, Michelle Bachelet, has pointed out, “the battle against this pandemic cannot be won if governments refuse to recognize the flagrant inequalities that the virus is bringing to the surface” [51]. Antonio Guterres, the Secretary General of the UN, pointed out in his intervention before the Human Rights Council that “the time for readjustment has come. Regarding remodeling and reconstruction, and from recovery to improvement, the call for a “global rethink” puts human rights at the center of all action [52]. This has been called by the UN as “recovering better”. In other words, take advantage of the crisis to overcome the structural barriers of inequality and exclusion, avoiding a return to the pre-pandemic situation. Along these lines, in addition to being a human rights issue, the TRC-COVID19 will serve to consolidate social development. Likewise, it will have a positive effect on ensuring the “Sustainable Development Goal 16”, which establishes that “conflicts, insecurity, weak institutions and limited access to justice continue to pose a serious threat to sustainable development” [53].

We became used to living with fear and uncertainty during the pandemic. We learned that having courage meant living with that uncertainty and dealing with the pain of losing our beloved ones. Only knowing the truth about what really happened, how it happened, and why it happened will allow us to build a future with an adequate ethical balance, where similar situations will never take place again.

This conceptual article is limited in scope and nature. We did not intend to analyze, from a legal perspective, the human rights violations that happened during the pandemic. Neither did we pretend to study specific national situations and the institutional challenges they had in the context of the pandemic. It was not our intention to analyze or systematize data or present case studies. The aim of this article is to rise questions and initiate a discussion on the possibility to use TJ as a tool to deal with the human rights violations and the institutional limitations Its practical implications should be subject to further debate and study. For instance, an immediate question that might rise is whether TJ application to the pandemic could be proposed under the wing of the United Nations, either the Office of the Hight Commissioner of Human Rights or the General Assembly. Other very practical issues such as funding, time frame, institutional size, among others, require future reflection.

## 5. Conclusions

The COVID-19 pandemic has exposed the weaknesses of health systems all over the world and has revealed the social inequalities that already exist. Through the mechanisms of TJ of truth, justice, reparation, and guarantees of non-repetition, societies seek to solve problems for the future. Its application in the post-pandemic context is feasible and can prevent the human rights violations documented during the pandemic from being repeated in similar future contexts. Specifically, this article focuses on suspension and restriction of human rights, and the use of state force, the limitations of the rights of women, corruption and human rights, and the impact on people in situations of vulnerability. A Truth and Reconciliation Commission should be established for the COVID-19 pandemic, in charge of governing the TJ process in a holistic, official manner and in connection with the victims of the given context. Recognition of the victims, civic trust, reconciliation, democracy, transparency, solidarity, and social inclusion are elements that will guide the TJ processes to achieve success and to face a better new normality.

## Data Availability

Not applicable.
